# Implications and Preventions of Cyberbullying and Social Exclusion in Social Media: Systematic Review

**DOI:** 10.2196/30286

**Published:** 2022-01-04

**Authors:** Adesoji Ademiluyi, Chuqin Li, Albert Park

**Affiliations:** 1 Department of Bioinformatics and Genomics University of North Carolina at Charlotte Charlotte Charlotte, NC United States; 2 Department of Software and Information Systems University of North Carolina at Charlotte Charlotte Charlotte, NC United States

**Keywords:** cyberbullying, cybervictimization, cyberaggression, bullying, mental health, social isolation, social media, mobile phone

## Abstract

**Background:**

The growth of social networking has created a paradigm in which many forms of personal communication are being replaced by internet communication technologies, such as social media. This has led to social issues, such as cyberbullying. In response, researchers are investigating cyberbullying to determine its implications in various life sectors.

**Objective:**

This manuscript reviews the methods, results, and limitations of the current cyberbullying research and discusses the physical and mental repercussions of cyberbullying and social exclusion as well as methods of predicting and counteracting these events. On the basis of the findings, we discuss future research directions.

**Methods:**

Using ScienceDirect, ACM Digital Library, and PubMed, 34 research articles were used in this review. A review was conducted using the selected articles with the goal of understanding the current landscape of cyberbullying research.

**Results:**

Studies have analyzed correlations between depressive and suicidal ideations in subjects as well as relationships in the social, educational, and financial status of the perpetrators. Studies have explored detection methods for monitoring cyberbullying. Automated detection has yet to become effective and accurate; however, several factors, such as personal background and physical appearance, have been identified to correlate with the likelihood that a person becomes a survivor or perpetrator of web-based cybervictimization. Social support is currently common in recovery efforts but may require diversification for specific applications in web-based incidents.

**Conclusions:**

Relations between social status, age, gender, and behaviors have been discovered that offer new insights into the origins and likeliness of cyberbullying events. Rehabilitation from such events is possible; however, automatic detection is not yet a viable solution for prevention of cyberbullying incidents. Effects such as social exclusion and suicidal ideations are closely tied to incidents of cyberbullying and require further study across various social and demographical populations. New studies should be conducted to explore the experiences of survivors and perpetrators and identify causal links. The breadth of research includes demographics from China, Canada, Taiwan, Iran, the United States, and Namibia. Wider ranges of national populations should be considered in future studies for accurate assessments, given global internet communication technology activity. The studies emphasize the need for formal classification terminology. With formal classification, researchers will have a more definite scope, allowing specific research on a single definable topic rather than on general bullying events and symptoms. Of all the studies, 2 used a longitudinal design for their research methodology. The low number of longitudinal studies leaves gaps between causation and correlation, and further research is required to understand the effects of cyberbullying. Research addressing ongoing victimization is required for the various forms of cyberbullying; social support offers the most effective current standard for prevention.

## Introduction

### Background

Internet communication technologies (ICTs) include a wide variety of platforms, ranging from social media, instant messaging, and chat rooms to email [1]; all of these affect our normal modes of communication [2]. The use of ICTs is increasing; meanwhile, negative consequences such as cybervictimization are being overlooked. Cybervictimization, colloquially *cyberbullying*, is a phenomenon proliferating through rising rates of interaction with social media [1]. Cyberbullying can be best defined as “an aggressive, intentional act carried out by a group or individual, using electronic forms of contact, repeatedly over time against a victim who cannot easily defend him or herself” [3]. This could be a distressing message about a victim’s appearance, delivered by a perpetrator over several web-based interactions with the purpose of delivering emotional or mental harm. Cyberaggression is formally defined as the intentional harm delivered by the use of electronic means to a person or group of people irrespective of their age who perceive such as offensive, derogatory, harmful, or unwanted [4]. One example is a mocking tweet regarding someone’s race or ethnicity, sent during a web-based interaction. Cyberaggression is inflicted on any individual and is a description of a singular incident as opposed to being repeated and targeted. Cyberbullying relates a cyberaggression event to a cybervictim, and correlates to a history of abuse where the opposing parties know each other on a personal level [5]. This implication does not hold for general cyberaggression [5]. As we are investigating cyberbullying and not cyberaggressions at large, it is important to recognize this distinction. Discourse surrounding cyberbullying is still new and has only seen consistent studies from 2007 to 2020 [3,5,6]. Researchers from fields such as sociology and psychology, now studying the phenomenon, struggle to classify it concisely because of the various forms it can take and its relation to traditional bullying. Several cyberbullying studies disagree regarding the overlap of cyberbullying and traditional bullying and use separate definitions to discern them from one another [5]. The tension stems from the assertion that cyberbullying is more soundly defined within the purview of cyberaggression [7]. Cyberbullying narrows the classification of instances of cyber-based attacks to those done over length of time; however, many in the field believe this is detrimental to the identification of events that may happen only once or between strangers [7]. In this review, we define cyberbullying and cyberaggression by their formal definitions and examine cyberbullying events on multiple occurrences.

Cybervictimization has a wide reach given the interconnectivity of each user [8]. Youth (ie, aged 11-18 years) are especially susceptible to this form of victimization [8] given the influence of peer interactions on social development in early life and conventional [9] standards. Several cases of suicide and suicidality and the presence of suicidal ideations and behaviors [10] have been found to have direct correlations to cyberbullying [11]. There are correlations between cyberbullying and mental health consequences, including depressive symptoms, particularly among youth and student populations [12]. Issues with mental health and social strain are also accumulating among college students and young adults, especially those in the age group of 18 to 24 years, who have screened 19% positivity for experiences of psychological distress because of cyberbullying incidents [10].

Social exclusion is a phenomenon that occurs when someone is forcibly or voluntarily separated from groups with whom they perform social interaction on a daily basis [13]. In clinical studies, social exclusion is associated with depressive symptoms and an increased risk of mortality [14]. It is possible that there are relations between the domains of cyberbullying, internet communications, and social exclusion, given an attack on a cybervictim being received through their preferred avenue of social interaction [1]. The likelihood of cyberbullying events among youth and adolescents suggests a considerable risk of social isolation to these populations [1]. Correlations between social isolation and workplace bullying have been drawn in adult studies [15]. These relations should be explored in school and in web-based environments so that a crisis among young people can be identified and prevented.

### Objectives

We review the current research in cyberbullying and mental health, its social outcomes, predictive factors, and novel approaches to management and suppression. We look to contribute a contextual understanding of cyberbullying unfound in the domain, make connections to social isolation and other sociological and psychological effects, and investigate methods to prevent the overall manifestation of these events. This analysis sheds light on current measures taken and future opportunities to combat the prevent the spread of cybervictimization.

## Methods

### Search Strategy

The primary electronic databases that were used in the review are Elsevier, ScienceDirect, PubMed, and ACM Digital Library. Each of these libraries is a globally recognized and reputable medical or scientific database, and they are ubiquitously used in medical and scientific research. Indexing terms for ScienceDirect and ACM Digital Library were used and were presented through the search as s*ocial isolation* OR *social exclusion* OR *social alienation* AND *cyberbullying* AND *social media*. MeSH terms were used for PubMed and were presented through search as *social isolation* OR *social exclusion* OR *social alienation* AND *cyberbullying* AND *social media*. These searches were conducted in accordance with the 2009 Preferred Reporting Items for Systematic Reviews and Meta-Analyses (PRISMA) guidelines. These searches were completed on February 23, 2020.

### Study Screening

This study’s scope covers cyberbullying factors in their relations to psychological and sociological disorders, social media, and intervening technologies. For these aims, a noted definition of cybervictimization sourced from an article by Smith et al [3] describes it as “an aggressive, intentional act carried out by a group or individual, using electronic forms of contact, repeatedly over time against a victim who cannot easily defend him or herself.”

**Figure 1:**
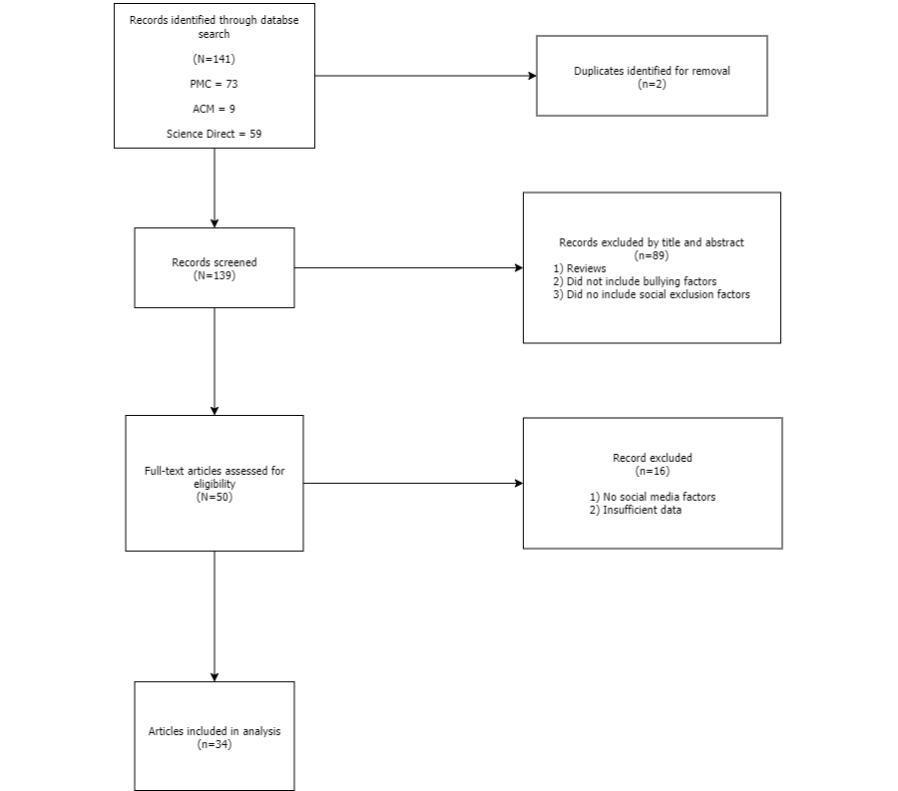
Screening flowchart.

The criteria for inclusion of the articles were defined by certain expectations. First, the articles were all original research studies that did not involve secondary reviews of underlying study categories. It was imperative that all studies involve primary data for accurate synthesis of the reviewed information. Second, all articles were required to include some form of cyberbullying factors and the effects they incur. Several articles only involved information about social exclusion or only information about social media and were excluded because they were unrelated to the primary topic. Conversely, it was imperative that all articles include social exclusion factors, given its relation to cyberbullying. Social exclusion factors relate specifically to the phenomena of social distance in reaction to a negative social event. The first screening required all articles to be nonreview, on the topic of bullying, and with factors relating to social exclusion. The articles were curated under these constraints and then screened further for 2 additional metrics. In the second screening, articles were excluded if they did not have significant social media factors, even if the included references to cyberbullying and social isolation, given that the research explicitly focuses on the sociological implications. Finally, the full-text screening involved a complete reading of the articles to determine the relevance of their findings. All records with insufficient information or data, such that they did not provide relevant or citable information, were excluded. The full screening is displayed in Figure 1.

## Results

### Landscape of Cyberbullying Research

The research surveyed from 2011 to 2020 focuses on the psychological effects of the phenomenon, with 10 of the 34 articles focusing on factors such as suicidal ideations, social agency, and depression [1,4,11,13,16-21]. Automated detection studies are studies that dealt with automated identification of cyberbullying events; this takes place through processing of text from logged chat room conversations [22]. A total of 3 articles dealt with methods of automated detection. Text filtering was a common strategy, using machine learning to perform sentiment analysis. Risk factor studies focus on characteristics that put someone at risk of cyberaggression [23]. Furthermore, 5 articles dealt with risk factors, such as age, oversharing tendencies, financial disposition, educational background, social media activity, and social status [1,23-26]. Predictive factors are behaviors that correlate with involvement in cyberbullying or cybervictimization, and 4 articles dealt with predictive factors of cybervictimization [17,24,27,28]. Predictive factors included parental status, access to goods, academic standings, and personal identity. Several articles focused on relationships between cyberbullying and traditional bullying, demographics, motivations, and information security. These articles provided information regarding the similarities and differences of web-based and in-person behavior and how they affect attitude and perception of events.

The articles dealt with several different ICT platforms, including Facebook, Twitter, Instagram, Tumblr, and many others. Articles that used a direct social media source aimed to discover behaviors and trends that could be used to map potential victims and perpetrators to quantifiable decisions and habits. Facebook and Twitter were the most common platforms used by researchers [29].

The research objectives are displayed in Table 1. The widest range of articles were related to psychological effects. These effects are often the focal point of research in cyberbullying. Predictive factors, risk factors, and demographic factors also have a substantial presence in the body of research. There is a smaller emphasis on technological applications in security awareness and automation throughout the research.

**Table 1:** Research objectives investigating cyberbullying.

Research objective	Study
Victimization risk factors	[1,23-26]
Perpetration predictive factors	[17,24,27,28]
Psychological effects	[1,4,11,13,16-21]
Security awareness	[1,28,30]
Automated detection	[22,31,32]
Cyberbullying demographics	[1,9,22,23,25,33-35]
Cyberbullying versus in-person bullying	[19,36]

### Forms of Cyberbullying

Limitations relating to the establishment of concrete definitions are an issue in cyberbullying research; however, a new body of research, which began in 2014, created new definitions for cybervictimization and differentiated it from traditional bullying [35]. Cyberbullying events can occur in many ways [34]. The most prominent methods are flaming, when a person sends angry or vulgar messages; harassment, when there is a consistent stream of offensive messages; denigration, when damaging messages about the target are sent to other associates of the target; masquerading, when the victim’s identity is stolen to imitate harmful or damaging messages produced by another entity; trickery, when the victim is deceived into providing personal information; exclusion, when a victim is ostracized from a social group [34]; stalking, when a person hacks or obtains personal information from a victim’s social media profile to determine their location and whereabouts; and blackmailing, when anonymous emails, telephone calls, and private messages are sent to a person to solicit money or actions from the victim [34].

### Demographics

The demographics in this review included children, adults, both genders, and various social groups. People of any age or status can be the subject of a cyberbullying incident [33]. Incidents present threats in several circles of interpersonal relations. Employed adults experience work position as a risk factor; 40% of people cyberbullied in the workplace are in some supervisory position [33]. Furthermore, 10% of working adults are survivors of cyberbullying [33]; however, adolescents between the ages of 13 and 21 years are more vulnerable to peer victimization [22]. Internet anonymity and the ability to interact with anyone anywhere pose severe risks [22]. Adolescents are using technological resources at the record rates [25]. Smartphones are the most common ICTs used by adolescents and can be accessed from any location with cellular service [1]. Large percentages of females are exposed to threatening messages (50%), stalkers (32%), requests for sexual information (27%), requests for self-sexual images (20%), and cyberbullying (17%) [1]. Adolescent males who had access to social media through ICTs became exposed to threatening messages (26%), requests for sexual information (15%), cyberbullying (14%), stalkers (14%), and requests for self-sexual images (10%) after use [1]. The variability in risks, such as substance abuse, self-harm, cybervictimization, and depressive symptoms, caused by these forms of exposure on various age groups is still new in academic study [1].

Studies suggest adolescent females are victimized at the highest rates [35]. They are more at risk in their teenage years than males, facing cyberbullying rates of 18% at the age of 13, 15% at 14, 24% at 15, and 21% at 16 [1]. Studies among college students showed that 44% of the female students reported experiencing some form of cybervictimization [35]. Cyberbullying is experienced by both males and females, yet there are many differences in the frequency of victimization that requires investigation.

University students reported that 50% of the cyberbullying they experienced was from classmates, 57% was from outside sources, and 43% was from unrelated individuals [34]. Cyberbullying has become a commonplace in higher education, with half of the college age students encountering it in some form. The investigated demographics are listed in Table 2.

Age, gender, and social status provided the most contextual information in the research. These factors may imply trends in cyberbullying.

**Table 2:** Demographics investigated by researchers.

Investigated demographics	Study
Gender	[1,35]
Social status	[33,34]
Age	[1,9,22,23,25,33]

### Causes

Predictive factors indicate where cyberbullying may occur. A factor may provide information about the perpetrator or the survivor. The literature identifies several factors that range from visual appearance to personal history. Studies have shown [4,23,24,37-39] several initiators of cyberbullying. The leading factor among cyberbullying perpetrators is the presence of normative thinking, peer pressure, and involvement in normatively aggressive peer groups [37]. The peer group is often the most influential group in a person’s social experience, especially among youth [37]. Low self-control and difficulty discerning moral identity are factors that allow peer pressure to inform a person’s actions [4]. When a social event is normalized in the peer group, the subject views the interaction as agreeable even when it contradicts pre-existing moral values [37].

An individual’s perspective on their performance affects their decisions, this is known as self-concept [19]. Self-concept is formally defined as “the relatively stable schemata of oneself that are generalized to the extent that they refer to an individual’s view of him- or herself across different situations” [19]. Social success is a salient indicator of happiness and a driver of behaviors [19]. In a study on psychological outcomes in social media interactions, a positive correlation was found between high self-concept and social success in peer groups [19]. An individual’s view of themselves is affected by environmental factors, such as household income, parental marriage status, gender, and their access to social resources and community [18,19,26]. In a study in Iran by Kabiri et al [4], poor performance in school and growing up in a low-income household also had positive correlations with perpetration likelihoods [4]. Another study of male boarding students corroborated the correlation of low-income students and cyberbullying [23]. These students are more likely to be perpetrators and have a higher vulnerability to peer victimization [24], given that early age interactions and social development may be limited when certain activities are prevented by a payment gap. Weak emotional bonds with parents and high discipline levels are common in perpetrators [26]. Both genders present similar likelihoods of becoming cyberaggressors [38].

Trends in the studies show the social behaviors of participants who reported receiving negative remarks, unwanted sexual suggestions or images, negativity from peers, humiliating targeted posts or had their accounts hacked [28]. The first major identifier was the tendency to post indiscreet information and content on social platforms without security [28]. This factor is positively associated with victimization likelihood and accounts for 18% variance in data [28]. There is a correlation between security and victimization incidents. Often, those who are victimized lack security on their social media profiles or are not equipped with the skills to implement security on their own [28]. A study by Saridakis et al [30] demonstrated that higher awareness of security risks and an ability to control generated information actively creates safer and more user-friendly environments, critical in preventing the likelihood of victimizing events.

Another contributing factor is facial features. A study on visual perception found that survivors of social ostracization (ie, exclusion by general consent from social acceptance of a group) are likely to be those whose faces are perceived as being incompetent and *cold* (unfriendly) [39].

Causes of cybervictimization events include factors of the environment, individual behavior, and ideations of the self and the environment. Several initiators complicate prediction and may need to be addressed individually.

A detailed list of studies related to causes of perpetration and victimization is shown in Table 3.

**Table 3:** Causes of cyberbullying perpetration and victimization events.

Causes	Study
Social pressures: peer grouping, social success	[4,19,37]
Web-based behavior: security awareness, social tendencies	[28,30]
Self-concept: identity development, social status, educational status	[19,37]
Public perception: perceived appearance, nonverbal interactions	[39]
Familial issues: marital status, home environment, parental relationships	[4,23,26]

### Effects of Cyberbullying

Effects are a crucial portion of the research because they contain results related to suicide and depression. Survivors of cybervictimization present high psychological distress, depression, and substance abuse [21]. This phenomenon is distinct from traditional bullying in its psychological effects [40]. Stress posting and oversharing are behaviors that often have a heavy correlation with cyberbullying [41]. These behaviors also cause the individual to become more likely to be targeted by a cyberbully [41] and suggest the possibility of cyclical processes. The scope of negative psychological outcomes of cyberbullying is summarized in Table 4.

**Table 4:** Negative psychological outcomes of survivors of cyberbullying.

Negative psychological effects	Study
Social exclusion	[13,18,19,32,39]
Self-harm	[1,10,21]
Depressive symptoms	[11,12,17,28,40,41]
Substance abuse	[10,17,21,26,40]

Social isolation, the experience of a person who has been ostracized from a social group, is a common effect of cybervictimization, as exclusion is a main tactic used in web-based cyberbullying [13]. When a person is ostracized, it is common for them to lose a sense of agency because of removal from the group that facilitated their social mobility [13]. Social agency is the feeling of control over one’s actions and the effects of those actions [13]. In some cases, bullies may use others within a social circle to isolate a person without directly involving themselves [32]. Facial features that fail to evoke feelings of empathy from viewers play a role in the likelihood of social exclusion [39]. High amounts of supplementary web-based communication have been closely associated with feelings of social isolation [19]. When a person has been socially ostracized, they find it harder to rally social support, as being isolated may corrode most of their social connections [18]. Still, it is uncommon for cyberbullying to spill over into real-world interactions. A study by Pabian et al [36] found cases often stay exclusively on the internet or offline.

Disadvantaged adolescents are often involved with the misuse of ICTs and become tangentially exposed to self-harm, substance abuse, and suicidal ideations [21]. These youth tend to develop habits of misusing personal data and neglecting social relationships and schoolwork, leading to sexual abuse, blackmail, threats, and, in some cases, the incitation of violence [1]. Events such as social exclusion, self-harm, substance abuse, and depressive symptoms were emphasized heavily in the literature.

### Methods of Prevention and Amelioration

There is limited research on approaches for deterring or recovering from cybervictimization. Social support is a generalized approach for social issues brought into the domain of cybervictimization. New research on technical prevention is scarce and have limited effectiveness. We found 3 studies that investigated potential methods for cyberbullying detection [22,31,42]. A study on the social networking site ASKfm provides research on a machine learning support vector machine classifier that detects instances of aggressive communication [22]. Experiments showed that 64% was the highest accuracy achieved by the algorithm [22]. A study by Ptaszynski et al [31] in 2016 found that natural language processing tested a 30% drop in performance over just a year of testing.

Cybervictimization incidents are embarrassing events, which can cause repression of experiences and discourage social support requests [43]. Social support is the tangible and intangible assistance from friends, partners, family members, and others [11]. Members in a community encourage and affirm an individual to stabilize their mental health and improve their self-concept [11]. Social support can occur on the internet or in-person. Web-based intervention is useful in isolation as it benefits those without in-person social support systems [11]. Assistance through social support occurs before or after an individual encounters cyberaggression [24]. Social support has produced reliable results in ameliorating cybervictimization effects [24]. Adolescents who perceived high levels of social support from family members were less likely to experience cyberbullying [24]. Social support actively reduces the effects of ongoing cyberbullying [11]. On average, males required more social support in these instances than females [24]. Multiple intersections of gender in the literature may prove to be a salient factor for future research. There were articles on investigating methods of prevention [22,31,42] and on investigating amelioration [22,31,42].

### Research Methods of Current Studies

We identified 4 research methods: exploratory, experimental, longitudinal, and cross-sectional. For this review, exploratory design is defined as research conducted in domains with little or no previous study. These studies are often foundational for future research and promote familiarity with the scope of the research. Experimental design is defined as an approach where the researcher has control of all the variables being manipulated and observed. The focus of these studies is accurately predicting and modeling an outcome based on a hypothesis. Longitudinal design is defined as a study that takes place with a recurring sample over a fixed length of time. Longitudinal studies focus on changes and patterns that develop over long time frames. Cross-sectional design does not rely on time and focuses on existing differences between sample members for one-time data collection. Cross-sectional studies were the most common, given quick access to information through population surveys. The study designs are displayed in Table 5.

There are 2 types of data analysis in the studies: qualitative and quantitative analysesQualitative analysis is defined as descriptions of specific situations; for example, the use of interviews, observations, and documents to describe things. Quantitative analysis is defined as data that are represented in numerical form, such as frequencies and averages: these are measurements. In this study, quantitative analysis was the most prominent. The analysis types by article are listed in Table 6.

**Table 5:** Designs of the reviewed studies.

Design	Study
Cross-sectional	[1,4,11,12,18,19,23,25-28,30,34,35,37,38,41,42,44,45]
Experimental	[13,16,20,31,32,43]
Longitudinal	[21,40]
Exploratory	[22,24,36,39]

**Table 6:** Methods of analysis in the reviewed studies.

Data type	Study
Quantitative analysis	[1,10-12,16,18-21,23-26,28,30-32,34,35,37-41,43,45]
Qualitative analysis	[4,16,22,27,36,42]

Throughout the review, important factors across the articles were determined. Multimedia Appendix 1 [1,4,10-13,16,18-28,31-44] summarizes salient information relating to the research processes used in each study. The author’s findings and limitations are summarized for reference. Methods for the deployment of individual studies were recorded as well as the target age demographics. Information relevant to the scope of the review discovered was recorded under social media factors.

## Discussion

### Principal Findings

Our review examines current articles relating to cyberbullying and identifies trends in perpetration and victimization. This contributes to the discussion of cyberbullying prevention approaches given the lack of sufficient technologies to censor it from its victims, likely perpetrators and victims, and consequences of their occurrence. The results provide predictive information relating to age, social status, and gender as well as information on types of cyberbullying, where they occur, and their effects.

We found various forms of cyberbullying in the literature that carry specific psychological effects. Gaps in research design limit the understanding of these events. Cyberbullying incidents carry serious mental health effects for victims leading to psychological disorders and suicidal ideations. New studies have shown that cyberbullying leads to real-world decisions, such as self-harm, abuse, and substance abuse [1,6,10]. Our review discerned information about the types of people and behaviors associated with cyberbullying victimization and perpetration. Most of those who are affected are adolescents and college students as well as children. Cyberbullying perpetration varies by age, social class, family life, and academic standing. The resources a person has in their community also affect the way someone can cope with being victimized. Cyberbullying is preventable and mitigatable. The most successful form of prevention of cyberbullying is robust social support systems, as technology cannot provide solid methods for counteraction in real time.

There is a lack in information on cybervictimization perpetrators because of the self-report nature of most surveys [23]. First, many involved in cyberaggression and victimization refuse to participate in studies even when anonymity is ensured by the facilitators [23]. Second, a wide margin of the survey-based studies used cross-sectional methods making it difficult to discern causation of the discovered effects [2,10,21,23,24,26,27,38]. It is not possible to make concrete causal links to behaviors without a long-term process [34]. Of the studies that used longitudinal methods, causal links, such as the direct relation to victimization and substance abuse, were discovered [21]. Third, given the emergent status of cybervictimization, there are various discoveries throughout the literature that reveal new factors not previously associated with cybervictimization that have value in its scope [33,39]. The impact of technologies on social relationships is likely an important factor relating to risk factors [33]. Fourth, there are significant limitations on the basis of demographics surveyed in many of the cross-sectional studies [18,42,46]. Cybervictimization is closely linked with social habits that may vary across different countries and in different social spheres [12,19,28,30,37,41,42]. Most of the literature focuses on participants above the age of 17; however, there are several indications that it may become rampant in children and teenage social spheres [1,12,17,23]. Trends in access to technology allow vulnerable age groups to access ICTs [1]. Research should focus on these phenomena using longitudinal design to interpret the unique issues in child cyberbullying. Finally, studies that included text monitoring systems did not experience acceptable success rates within their testing periods [22,32,42]. High rates of accuracy are required for filtering methods to be efficient; among the studies, 65% accuracy was the highest rate produced given the limitations in syntactic nuance [22]. Syntactic analysis of aggressive interactions is difficult to discern from healthy interactions given disparate standards of communication across web-based platforms [22]. Research on language in insular internet communities should be investigated to expand understandings of web-based communication.

### Forms of Cyberbullying

Among the types of cyberbullying (flaming, harassment, denigration, masquerading, trickery, exclusion, stalking, and blackmailing), some give rise to dangerous effects, such as social isolation and suicidal ideations. Information on the frequency of the various types of cyberbullying events should be investigated to determine the implications of each form. Research should be driven on the forms that have the highest correlation to suicidality and depressive symptoms. Behaviors that lead to physical harm and death require the quickest responses.

### Demographics

Recent reviews of cyberbullying literature lack detailed information on salient factors in predicting and preventing its occurrence in youth and adults. Given that cyberbullying is beginning to be seen in places such as elementary schools, with as high as 85% rates of web-based messaging use in preteen populations, research is crucial to assure the mental health of younger students. Adults are less vulnerable; yet, more research is required to fully understand the complex relations in the workplace surrounding cybervictimization [23]. Gender may play a role in the likelihood of victimization, as females see higher rates of exposure to cyberbullying [1,35]. Females receive more consistent support than males [11]. Peer groups and setting plays a large role in determining if someone may cyberbully or be victimized. Developing children are exposed to fluctuations of these dimensions of their social experience before understanding the complexities of social interactions [9]. Counseling resources are effective in handling distress in cyberbullying event. Those who are likely to be victimized share information at high frequencies on social media accounts, have high engagement levels with social media platforms, and have very little understanding of the importance of personal information security [23,28,30]. This emphasizes the need for research on young children with access to ICTs.

### Negative Mental Health Outcomes

Cybervictimization can result in negative mental health outcomes, including depressive symptoms, suicidal ideations, and substance abuse, which are prevalent for young adults and adolescents between the ages of 15 and 23 [10]. These victimization events cause depression and can lead to the deterioration of self-concept and academic performance of students. Social exclusion, a process where individuals are excluded from their social circles, can cause them to lose their perceived agency and expediate other negative mental processes [13]. This can result in unhappiness that drives victims to depend on web-based interactions for social experience [12]. The dangers as well as mental abuse, sexual abuse, and drug use are even more of a threat to younger victims as they will have less utility to navigate social complexities than their older counterparts [1]. This is problematic in a time when intentional studies prove that victimization events on occasion escalate to face-to-face interactions and altercations [1,8].

### Effective Cyberbullying Prevention

The effects of cyberbullying can be ameliorated by social support [11]. Web-based social support is effective for those who lack strong social connections [11]. College students and working age adults who are away from family environments for work or school could benefit from these web-based systems. Children and adolescents whose families are present in day-to-day life benefit more from in-person social support intervention, where development of communication and support practices occur through pre-existing relationships. One challenge for social support outreach is that social ostracization can cause victims to lose their sense of agency and steer away from forms of social support [24]. Negative feelings associated with cyberbullying can cause victims to feel incapable of expressing their experiences and suppress them [43]. During these events outreach can only be initiated if the victim is willing to divulge information about the instance. Given the volatility of events after initiation and the tendency for victims to become isolated, preventive measures are key in protecting the mental stability of the victim [13].

The current methods of automatic detection [22,31] struggle with nuance in web-based communications. Ptaszynski et al [31] extracted phrases and categorized them by harmfulness, based on seed words detected in specific phrases. This study used syntactic positioning of words to determine harmfulness within the messages [31]. This approach achieved up to 90% accuracy but dropped in performance because of the limitations in further data extraction and issues categorizing nonharmful phrases [31]. Future models should continue optimization and include nonharmful entries and neutral phrases. Van Hee et al [22] used ASKfm training data sets for phrase annotation with the objective of classifying the role of the participants of the cyberbullying event and the type of cyberbullying that occurred. This approach achieved up to 64% accuracy but lacked context for concise classification of cyberbullying types and could not accurately determine participant roles [22]. A new model that detects sentiments of the victim, rather than incitement from the perpetrator should be studied for more accurate determination of cyberaggression [22]. The language of different social groups can take many forms in web-based discourse and can range a wide spectrum of literal and coded speech that is rarely clear cut for unexperienced readers. The model should identify speech patterns on the basis of a sizable history of interaction to make accurate predictions rather than simply classifying based on detected words and phrases.

Early intervention is a potential approach for prevention. Research should be headed to investigate the effects of poverty, social stressors, parent marital status, and environment on internet behaviors and tendencies [4,37]. Cyberbullying can be combated by intervention, with different methods for web-based spaces. Given additional data, victim prevention and treatment can be improved. Research on how cyberbullying effects specific geographic regions, ethnic groups, and age ranges [1,18,21,23] should also be continued; these factors may often determine the psychological outcomes of the victim. New research should offer new perspectives for preventing the proliferation of cyberbullying and social isolation.

There are many technologies that are in development today that could be beneficial if applied to the study and prevention of cyberbullying [22,31,32,39]. Natural language processing is widely studied today and can be the basis for understanding and preventing cyberbullying [32,42]. Methods of recovery are also being studied through social support programs [24]. Recovery efforts are common for general depression and anxiety and should be improved to focus on issues specifically related to cyberbullying. An example of this is social support programs that promote emotional health [11]. Similarly, a method of deployment of social support programs to isolated individuals using technology should be further investigated to provide for the event of social isolation caused by cyberbullying

### Limitations

The review was limited to 3 specific databases, therefore information about surveys and other research studies on other major and minor databases is excluded from this review. This narrows the scope of information available for consideration in the review to the largest and most beneficial database but may omit potentially useful granular data. The search did not include any articles that lacked information specific to cyberbullying, this means that information pertaining to more general bullying studies that proved pertinent was included. Articles on young children were limited in data about social phenomena influencing behaviors relating to ICTs, making it more difficult to assess the relationship between young children and cyberbullying trends. More longitudinal studies would assist in the understanding of perpetrators and the relations of victim likelihood within adult and child populations [26]. In future research, longitudinal studies are required [26] for tracking cyberbullying victims and perpetrators to support a comprehensive evaluation of their behaviors and outcomes.

### Conclusions

Cyberbullying is a newly emerging phenomenon that has proliferated through the global rise of ICTs that began to converge internationally between 2000 and 2008 [2]. Previous reviews have highlighted the severity of the phenomenon [6,47,48], yet do not address solutions combating the rapid advance of cybervictimization in the social media era. To confront cybervictimization in the social media era, reevaluation of factors in the scope of the current research and longer-term longitudinal studies for causal links to be ascertained regarding suicidal or depressive symptoms is required. A larger emphasis on demographic groups should be taken to make clear determinations about how cyberbullying effects people of varying age, race, gender, and economic class. Ho et al [2] noted a wide range of ICT activity across resource-limited and transitional countries; however, few countries have been explicitly studied. Future research should be carried out in more geographic locations, as it requires holistic representation of disparate racial and gender populations. Relations between cyberbullying and predictive factors, such as low socioeconomic status, gender, and the presence of divorce, were identified in addition to studies drawing associations between cybervictimization and mental health outcomes, such as depressive and suicidal ideations. Ideations have been shown to lead to lower academic performance, retaliatory action, and suicide. Detection is a relevant method of counteracting the effects of cyberbullying on youth and adult populations and needs consistent research to keep pace with the rate of ICT growth. Detecting instances of cyberaggression is a challenging process given the nuances of web-based communication and the self-report nature of events [12]. Discovery of ongoing victimization incidents is necessary to reach current victims of cyberbullying, while predictive factors and preventive measures are required to halt future growth.

## Appendix

Multimedia Appendix 1 Summary of individual studies.

## Abbreviations

ICT: internet communication technology

## References

[1] Maoneke P, Shava F, Gamundani A, Bere-Chitauro M, Nhamu I (2018). ICTs use and cyberspace risks faced by adolescents in Namibia. Proceedings of the Second African Conference for Human Computer Interaction: Thriving Communities.

[2] Doong SH, Ho S (2012). The impact of ICT development on the global digital divide. Electron Commer Res Appl.

[3] Smith PK, Mahdavi J, Carvalho M, Fisher S, Russell S, Tippett N (2008). Cyberbullying: its nature and impact in secondary school pupils. J Child Psychol Psychiatry.

[4] Kabiri S, Shadmanfaat S, Choi J, Yun I (2020). The impact of life domains on cyberbullying perpetration in Iran: a partial test of Agnew's general theory of crime. J Crim Justice.

[5] Olweus D, Limber SP (2018). Some problems with cyberbullying research. Curr Opin Psychol.

[6] Bottino SM, Bottino CM, Regina CG, Correia AV, Ribeiro WS (2015). Cyberbullying and adolescent mental health: systematic review. Cad Saude Publica.

[7] Corcoran L, Guckin C, Prentice G (2015). Cyberbullying or cyber aggression?: a review of existing definitions of cyber-based peer-to-peer aggression. Societies.

[8] Donegan R (2012). Bullying and cyberbullying: history, statistics, law, prevention and analysis. Elon J Undergrad Res Commun.

[9] Coley R, Spielvogel B, Kruzik C, Miller P, Betancur L, Votruba-Drzal E (2021). Explaining income disparities in young children’s development: the role of community contexts and family processes✰. Early Child Res Q.

[10] Cénat JM, Smith K, Hébert M, Derivois D (2019). Cybervictimization and suicidality among French undergraduate students: a mediation model. J Affect Disord.

[11] Cole DA, Nick EA, Zelkowitz RL, Roeder KM, Spinelli T (2017). Online social support for young people: does it recapitulate in-person social support; can it help?. Comput Human Behav.

[12] Williford A, Orsi R, DePaolis KJ, Debbie I (2018). Cyber and traditional peer victimization: examining unique associations with children’s internalizing difficulties. Child Youth Serv Rev.

[13] Malik RA, Obhi SS (2019). Social exclusion reduces the sense of agency: evidence from intentional binding. Conscious Cogn.

[14] Cacioppo JT, Cacioppo S, Capitanio JP, Cole SW (2015). The neuroendocrinology of social isolation. Annu Rev Psychol.

[15] Einarsen S, Hoel H, Notelaers G (2009). Measuring exposure to bullying and harassment at work: validity, factor structure and psychometric properties of the Negative Acts Questionnaire-Revised. Work Stress.

[16] Kim J, Shim H, Hay C (2020). Unpacking the dynamics involved in the impact of bullying victimization on adolescent suicidal ideation: testing general strain theory in the Korean context. Child Youth Serv Rev.

[17] Pantic I (2014). Online social networking and mental health. Cyberpsychol Behav Soc Netw.

[18] Wang C, Chang Y, Yang Y, Hu H, Yen C (2019). Relationships between traditional and cyber harassment and self-identity confusion among Taiwanese gay and bisexual men in emerging adulthood. Compr Psychiatry.

[19] Khan S, Gagné M, Yang L, Shapka J (2016). Exploring the relationship between adolescents' self-concept and their offline and online social worlds. Comput Human Behav.

[20] Birk MV, Buttlar B, Bowey JT, Poeller S, Thomson SC, Baumann N, Mandryk RL (2016). The effects of social exclusion on play experience and hostile cognitions in digital games. Proceedings of the 2016 CHI Conference on Human Factors in Computing Systems.

[21] Cénat J, Blais M, Lavoie F, Caron P, Hébert M (2018). Cyberbullying victimization and substance use among Quebec high schools students: the mediating role of psychological distress. Comput Human Behav.

[22] Van Hee C, Jacobs G, Emmery C, Desmet B, Lefever E, Verhoeven B, De Pauw G, Daelemans W, Hoste V (2018). Automatic detection of cyberbullying in social media text. PLoS One.

[23] Li J, Sidibe AM, Shen X, Hesketh T (2019). Incidence, risk factors and psychosomatic symptoms for traditional bullying and cyberbullying in Chinese adolescents. Child Youth Serv Rev.

[24] Shaheen AM, Hamdan KM, Albqoor M, Othman AK, Amre HM, Hazeem MN (2019). Perceived social support from family and friends and bullying victimization among adolescents. Child Youth Serv Rev.

[25] Lareki A, Martínez de Morentin JI, Altuna J, Amenabar N (2017). Teenagers' perception of risk behaviors regarding digital technologies. Comput Human Behav.

[26] Livazović G, Ham E (2019). Cyberbullying and emotional distress in adolescents: the importance of family, peers and school. Heliyon.

[27] Young R, Len-Ríos M, Young H (2017). Romantic motivations for social media use, social comparison, and online aggression among adolescents. Comput Human Behav.

[28] Kokkinos CM, Saripanidis I (2017). A lifestyle exposure perspective of victimization through Facebook among university students. Do individual differences matter?. Comput Human Behav.

[29] Laranjo L (2016). Social media and health behavior change. Participatory Health Through Social Media.

[30] Saridakis G, Benson V, Ezingeard J, Tennakoon H (2016). Individual information security, user behaviour and cyber victimisation: an empirical study of social networking users. Technol Forecast Soc Change.

[31] Ptaszynski M, Masui F, Nitta T, Hatakeyama S, Kimura Y, Rzepka R, Araki K (2016). Sustainable cyberbullying detection with category-maximized relevance of harmful phrases and double-filtered automatic optimization. Int J Child Comput Interac.

[32] Sánchez-Medina AJ, Galván-Sánchez I, Fernández-Monroy M (2020). Applying artificial intelligence to explore sexual cyberbullying behaviour. Heliyon.

[33] Forssell R (2016). Exploring cyberbullying and face-to-face bullying in working life – prevalence, targets and expressions. Comput Human Behav.

[34] Peled Y (2019). Cyberbullying and its influence on academic, social, and emotional development of undergraduate students. Heliyon.

[35] Selkie EM, Kota R, Moreno M (2016). Cyberbullying behaviors among female college students: witnessing, perpetration, and victimization. Coll Stud J.

[36] Pabian S, Erreygers S, Vandebosch H, Van Royen K, Dare J, Costello L, Green L, Hawk D, Cross D (2018). “Arguments online, but in school we always act normal”: the embeddedness of early adolescent negative peer interactions within the whole of their offline and online peer interactions. Child Youth Serv Rev.

[37] Ho SS, Chen L, Ng AP (2017). Comparing cyberbullying perpetration on social media between primary and secondary school students. Comput Educ.

[38] Beckman L, Hagquist C, Hellström L (2013). Discrepant gender patterns for cyberbullying and traditional bullying – an analysis of Swedish adolescent data. Comput Human Behav.

[39] Rudert S, Reutner L, Greifeneder R, Walker M (2017). Faced with exclusion: perceived facial warmth and competence influence moral judgments of social exclusion. J Exper Soc Psychol.

[40] Landoll R, La Greca AM, Lai B, Chan S, Herge W (2015). Cyber victimization by peers: prospective associations with adolescent social anxiety and depressive symptoms. J Adolesc.

[41] Bunney P, Zink A, Holm A, Billington C, Kotz C (2017). Orexin activation counteracts decreases in nonexercise activity thermogenesis (NEAT) caused by high-fat diet. Physiol Behav.

[42] Blackwell L, Dimond J, Schoenebeck S, Lampe C (2017). Classification and its consequences for online harassment: design insights from HeartMob. Proc ACM Hum Comput Interact.

[43] Horner S, Asher Y, Fireman GD (2015). The impact and response to electronic bullying and traditional bullying among adolescents. Comput Human Behav.

[44] Lev-On A (2017). The third-person effect on Facebook: the significance of perceived proficiency. Telemat Inform.

[45] Camacho S, Hassanein K, Head M (2018). Cyberbullying impacts on victims’ satisfaction with information and communication technologies: the role of Perceived Cyberbullying Severity. Inform Manag.

[46] Walker CM, Sockman BR, Koehn S (2011). An exploratory study of cyberbullying with undergraduate university students. Tech Trends.

[47] Hutson E, Kelly S, Militello LK (2018). Systematic review of cyberbullying interventions for youth and parents with implications for evidence-based practice. Worldviews Evid Based Nurs.

[48] Zych I, Ortega-Ruiz R, Marín-López I (2016). Cyberbullying: a systematic review of research, its prevalence and assessment issues in Spanish studies. Psicología Educativa.

