# Supplementary material for: Implications and Preventions of Cyberbullying and Social Exclusion in Social Media: Systematic Review
Source: JMIR Form Res. 2022 Jan 4;6(1):e30286. doi: 10.2196/30286 (PMC8767467; doi:10.2196/30286)
Supplement: Multimedia Appendix 1 [file formative_v6i1e30286_app1.docx]

**Appendix A** Summary of individual studies.

| Article title | Age group | Methodology | Social media factors | Primary findings | Author’s reported limitations |
| --- | --- | --- | --- | --- | --- |
| ICTs^a^ use and cyberspace risks faced by adolescents in Namibia [1]. | Teenagers or adolescents (11-18 years). | Data were collected through a survey call of 729 participants. | Discovers use information about information communication technologies used by youth in Namibia. Information is revealed about social media perceptions and use. | The aim is to form better understanding of the risks of web-based interactions (through ICTs) associated with different age and gender groups, the particular focus group being adolescents. The studies reveal tangential information like the lack of access to ICTs in rural areas. Adolescents present interest in educational information. Females are presented with the biggest risk related to stalking and sexual harassment. Many youths use social media as a means of meeting strangers, and parents underestimate the risks associated with cyberspace interactions. | (1) Study is based on various time frames and frequency of misconduct, making results harder to generalize. |
| The impact of life domains on cyberbullying perpetration in Iran: A partial test of Agnew’s general theory of crime [4]. | Teenagers or adolescents (11-18 years). | 785 subjects self-reported in Iran. Selected individuals were interviewed about experiences in site. Data was analyzed through ordinary least square regression predicting models. | Subjects are called to self-report their involvement in cyber victimization incidents on social platforms. | Researchers hoped to investigate the relationship between several life domains and peripheral cyberbullying. Low self-control and moral identity were found to increase likelihood of cyberbullying perpetration. Self, family, and school domains are all effect by cyberbullying and negatively correlate with the increase of cybervictimization incidents. The study confirms previous studies. | (1) Not all emotions, such as anger, shame, and guilt were considered (2) Moderating factors other than self-esteem need to be examined to provide better policy implications for preventing adolescent suicide problems (3). Does not investigate power dynamics between bullies and victims (4). Capture emotional traits rather than emotional states (5).Generalizability of results may be limited by single-country sampling, may be regional differences (6). Females are excluded from the study, and gender may be a significant factor. |
| Cybervictimization and suicidality among French undergraduate Students: A mediation model [10]. | Young adults (18-25 years). | 4626 French undergraduate students are sampled and answered scaled questions. | The study investigates the results of negative web-based interactions such as cyberbullying. | The study examines the effects of cyber victimization and looks to understand how severe social and mental implications of cyberbullying are. Cybervictimization was found in higher rates in male students. Both genders suffered from suicidal ideations and attempts. | (1) Cross-sectional design makes it hard to observe developmental trajectories of cyber victimization (2). More comprehensive questionnaire would be beneficial in accessing various forms of cybervictimization and their impacts. |
| Perceived social support from family and friends and bullying victimization among adolescents [24]. | Teenagers or adolescents (11-18 years). | Study is a cross section of 437 adolescents. Personal experiences are marked as experienced or not in a checklist. | Cyberbullying as well as traditional bullying are considered in their effects of subjects of the study. | The study finds that perceived social support from family members significantly predicts lower bullying victimization. It is also shown that male adolescents need more support to reduce similar bullying victimization seen by female counterparts. | (1) Self-reporting (2) Cross-sectional (3) Only adolescents who were attending public schools. |
| Cyber and traditional peer victimization: Examining unique associations with children’s internalizing difficulties [12]. | Children (5-13 years). | Sample of 841 students from elementary schools is surveyed. | Web-based and traditional cyberbullying are compared. | Study hopes to analyze the negative consequences of cyberbullying (cybervictimization) and traditional bullying in youth. The study prefaces that younger children have a growing exposure to ICTs and growing rates. Although traditional bullying resulted in poor outcomes, cyber victimization did not produce any relevant symptoms. | (1) Phenomena unfold over time and longitudinal studies are needed to full disentangle effects from those that are traditional. Especially relating to new emerging technologies for information collection (2). Uses a modified version of the normative cybervictimization scale. More robust measures may capture this information in younger children. |
| Comparing cyberbullying perpetration on social media between primary and secondary school students [37] | Mixed | 1424 children were surveyed on a national scale. Students rated their attitudes and norms via the 4 dimensions by Heriman* and the 6 measures by Ajzen. | Students surveyed on their experiences with cyberbullying perpetration and web-based victimization. | The study aims to discover rates of cyberbullying perpetration in young children and teenagers. Social attitudes and normative behavior were the highest predictors of cyberbullying. Parental involvement significantly cut cyberbullying perpetration, with age having little effect. | (1) Did not examine other potential factors, such as perceived risks of being caught which may cause unethical behaviors (2). Cross-sectional, good for correlation but bad for causal relations. (3) Uses specific forms of mediation where parents may use other methods not discussed in the study. (4). Did not investigate social media consumption of psychological effects. |
| Unpacking the dynamics involved in the impact of bullying victimization on adolescent suicidal ideation: Testing general strain theory in the Korean context [16]. | Teenagers or adolescents (11-18 years). | Cross-sectional data involves 10,453 students in South Korea. Descriptive statistics were extracted and analyzed. | The scope includes bullying for both traditional and cyber environments. | The goal of the researchers is to explore the dynamics involved in relationships between bullying and suicidal ideations. It is discovered that the effects of negative emotions are decreased with personal esteem being high. There are direct and implied correlations between negative emotions and victimization/suicidal ideations. | (1) The research was limited to only student populations.(2) Certain attributes that were important in the scope of the study were excluded given data limitations. |
| Relationships between traditional and cyber harassment and self-identity confusion among Taiwanese gay and bisexual men in emerging adulthood [18]. | Teenagers or adolescents (11-18 years). | Cross-sectional survey is deployed at the national level. | Adolescents are surveyed for the uses and effects of social media activity. | Study attempts to uncover the motivations for social media use in adolescents. It found that social comparison is common and related to web-based aggressive victimization. Entertainment motivations trend away from social aggression while romantic motivations positively correlate. | (1) Cross-sectional research design limited capabilities determine casual relationships between experiences and self-identity confusion. (2) Self-reporting was used an additional information about victims could not be collected. (3) No follow-up with participants |
| Exploring the relationship between adolescents’ self-concept and their offline and web-based social worlds [19] | Mixed | 282 males and 451 females surveyed. Classes were visited and samples of students were given questionnaires. | Teenagers surveyed on their online and offline lives and self-conceptions web-based social interaction is a primary concept. | It was found that self-concept levels correlated with levels of socialization in both online and offline platforms. The positive correlations apply to those only highly socializing on web-based platforms. The study suggests that social success is the greatest predictor of contentment with social self-concepts. | (1) Self-concept was the sole indicator of well-being, not external evaluation. (2) Limited number of predictors and outcomes in the data. |
| Social exclusion reduces the sense of agency: evidence from intentional binding [13]. | Young adults (19-25 years). | Students are surveyed. The experiment used computer generated stimuli to test sensual and emotional awareness. | Deals primarily with social exclusion. Considers cyberbullying as a source of the underlying issues. | Given the negative effects of social exclusion, cyberbullying, and cyber ostracism, researchers wish to discover how social exclusion affects a victim’s mental state. The study reveals that the victim’s sense of agency and control are acutely affected when the victim is isolated. Victim becomes resigned. | —^b^ |
| The effect of social exclusion on play experience and hostile cognitions in digital games [20]. | Teenagers or adolescents (11-18 years). | Uses operator terms to simulate or induce feelings of social exclusion and model them. Participants are tested in multiple environments and then faced with ambiguous tasks that later are psychoanalyzed. | Web-based social interactions and social outcomes are observed through the purview of social exclusion. | The researchers in this study wanted to understand how hostile experiences in web-based video games develop, using social exclusion as the primary factors of dissonance between players. Exclusion predicted hostile cognitions; hostile cognitions also increased over time. | (1) Subjects were paid for participation, were not players interested in the games under their own volition.  (2) The link between hostile cognitions and exclusion is not unique to video games  (3). There may be positive outcomes that result from social ostracism. |
| Cyberbullying victimization and substance use among Quebec high-school students: the mediating role of psychological distress [21] | Teenagers or adolescents (11-18 years) | 3 waves of surveys recorded 1540 student participants. Each wave measured different stressors (psychological distress, substance use, interparental violence) | The study focuses on effects of substance abuse and family structures on web-based social abuse and cyber victimization | Researchers looked to find which unconventional factors affected the cyberbullying rates of Quebec youth. Gender and family violence have significant correlations to psychological distress. There is also a direct effect on cyberbullying victimization during substance abuse use periods, though likely a partial mediation effect | (1) Self-reported (2) Did not evaluate various types of cyberbullying experiences, focusing on scopes. |
| Automatic detection of cyberbullying in social media text [22]. | Teenagers or adolescents (11-18 years). | Data collected from social networking site *ASKfm.* Using a machine learning method based on linear SVM classifiers to assist human curation. | Social media platforms are used as the basis for text samples cyberbullying data collection. | The goal of the researchers was to essentially explore cyberbullying detection technologies as an alternative to manual monitoring. The machines returned 64% accuracy for English and 58% accuracy for Dutch. | —^b^ |
| Incidence, risk factors, and psychosomatic symptoms for traditional bullying and cyberbullying in Chinese adolescents [23]. | Teenagers or adolescents (11-18 years). | A cross-sectional study of 3774 participants is analyzed. | The study compares statistical information about traditional and cyberbullies. | The study attempts to explicate relations between traditional and cyberbullies and how cyberbullying and traditional bullying effects its victims. Male boarding students who have low academic performance are the most likely too bully. The study discovers psychosomatic symptoms are common in bullying victims. | (1) Unable to implement random sampling of schools, which can lead to section bias. (2) Self-report may explain why perpetration was seen in lower rates than victimization. |
| Exploring cyberbullying and face-to-face bullying in working life—prevalence, targets, and expressions [33]. | Adults (>25 years). | 400 respondents surveyed; participants given questionnaire via internet list. | Compares web-based and face-to-face bullying in the workplace environment. | Researchers hopes to find models in which cyberbullying is found in adult environments. It was found that supervisors were often the victims of cyberbullying rather than other employees. Men were more exposed the be behavior than women, however this was only true on the internet. Furthermore, 9.7% of respondents were bullied. | (1) There needs to be a clearer conceptual definition of cyberbullying. (2) Some behaviors may simply be regular or expected youth behaviors. |
| Teenagers’ perception of risk behaviors regarding digital technologies [25] | Teenagers or adolescents (11-18 years) | Data was analyzed from previous studies and recontextualized. 1486 youth were surveyed | Teenagers are surveyed to understand their perceptions of risk associated with social networking and digital technologies | The study attempts to understand the teenage perceptions of risk related to social media. It discovers that teenagers have high perceptions of risk behaviors relating to posting photos and data. There are differences found in age and sex, and it suggests that it may be necessary for training to be administered, especially to boys of older ages | —^b^ |
| Cyberbullying and emotional distress in adolescents: the importance of family, peers, and school [26]. | Young adults (19-25 years). | 259 young adults were surveyed with Likert-scale questionnaires. Data was analyzed using the General Aggression Model. | Adolescents are surveyed for their background data in relation to cyberbullying activities and relations. | The researchers examined the socioeconomic status, family, parenting styles, peer relations, and school factors to find correlations in the samples. It was discovered that girls are more likely to communicate cyberbullying events than boys. Students with lower academic achievement cyberbully more often. Traditional protective factors correlate negatively with cyberbullying in risk behavior etiology. | (1) Small research sample  (2) Parenting styles scales were of limited scope.  (3) Over and underreporting characteristics cannot be determined.  (4) Cross-sectional design does not reveal whether bullying victims attack other because of retaliation or become victims because of their own aggressive behavior. |
| Romantic motivations for social media use, social comparison, and web-based aggression among adolescents [27]. | Teenagers or adolescents (11-18 years). | Cross section’s survey is deployed at the national level. | Adolescents are surveyed for the use and effects of social media activity. | Study attempts to uncover the motivations for social media use in adolescents. It is found that social comparison is common and related to web-based aggressive victimization. Entertainment motivations trend away from social aggression although romantic motivations positively correlate. | (1) Cross-sectional nature of studies only allows associations between known variables.  (2). Uses web-based panel that did not account for network positioning and patterns.  (3). Does not account for sexual orientation, which can be an increased risk factor. Longitudinal study would increase accuracy. Motivations for social media use could be studied for additional context for correlations with risk. |
| A lifestyle exposure perspective of victimization through Facebook among university students. Do individual differences matter [28]? | Adults (>25 years). | 240 students using Facebook in Greece were sampled. Data gathered through questionnaire. Translated into English for accurate representations of conversion scale. | Facebook data collected from subject accounts to determine behavioral patterns between web-based victimized subjects. | The researchers hope to find conclusive evidence that the victim’s personal behavior should be considered when victimization is studied. The studies reveal that personal behaviors and risky web-based behaviors have a correlation to victimization. Depressive symptoms coupled with self-disclosure was a predictive factor for Facebook victimization. | (1) Study only includes students at Greek universities and may not be suitably generalized.  (2) Self-report measures may allow bias toward social desirability. |
| Cyberbullying behaviors among female college students: Witnessing, perpetration, and victimization [35] | Young adults (~18+ years). | Web-based surveys of the participants from 4 colleges. 11 behaviors investigated. | Data is collected from students based on experience in web-based social interactions. | The goal of the researchers is to investigate the severity of cyberbullying in female students in college environments. 44% of participants in the study experienced cyberbullying, almost half of the surveyed individuals. Cyberbullying is common among college women, though much of the experienced cyberbullying is witnessed, implying smaller groups of victims and larger groups of passive observers. | (1) There was a very limited research scope and given the cross-sectional nature of the research, long-term analysis could not be used.  (2). The sample was largely ethnically homogeneous and small and may have excluded valuable experiences of other groups. |
| Cyberbullying and its influence on academic, social, and emotional development of undergraduate students [34]. | Young adults (19-25 years). | 638 Israeli students were surveyed from 32 item questionnaire. Frequency is tested on a 5-item scale. | Surveys students to discover experiences of social media. | Researchers desired to form an understanding of how cyberbullying affects academic and social aspects of affected students. Over half of the test subjects were found to have experienced some form of cyberbullying on multiple occasions. Social messaging was the predominant source of cyberbullying. The results suggest that there is an influence on academic and social health, but conclusions are inconclusive. It is noted that religion may be a predicting factor. | —^b^ |
| Discrepant gender patterns for cyberbullying and traditional bullying—an analysis of Swedish adolescent data [38]. | Teenagers or adolescents (11-18 years). | Swedish students surveyed from national survey of 3000 participants. | Students surveyed for personal and survey data regarding their relationships to technological devices and social media platforms, and cyberbullying victimization. | Researchers looked to find the gender differences in cyberbullying interactions. Both genders had similar likelihoods of becoming bullies, yet girls were more likely to be victims than boys. | —^b^ |
| Faced with exclusion: Perceived facial warmth and competence influence moral judgments of social exclusion [39]. | Adults. | Images are manipulated and participants select desirable manipulations. | The scope of effected environments includes internet platforms and web-based social outlets or in online gaming environments. | The researchers set out to investigate how facial appearance may contribute to social exclusion through psychological biases. Ostracizers are imagined cold and incompetent looking, and these features are generally selected for social exclusion. | —^b^ |
| Individual information security, user behavior, and cyber victimization: An empirical study of social networking users [30]. | Mixed. | Data extracted through surveys from 400 respondents. Survey participants were selected based on diversity. Respondents were asked about cybercrime and their experiences. | Respondents surveyed on their perceptions of social harassments, cybercrime, spam, fraud, and harassment. | Researchers look to understand the relationships between information security on SNS services and web-based victimization. A negative association between dominant SNS language and victimization as well as the perception of information control via SNS. | (1) Only measures victimization that is known to users. |
| Cyber victimization by peers: Prospective associations with adolescent social anxiety and depressive symptoms [40]. | Adolescents (14-18 years). | 839 students sampled twice once every 6 week. Used *The Revised Peer Experiences Questionnaire* to assess peer victimization. | Surveys are administered to victims of web-based cybervictimization. | Researchers hope to investigate the short-term relationships between cyberbullying and social anxiety in adolescents. Cyberbullying was found to be distinct from other forms of peer victimization and was associated with increased levels of depressive symptoms. | (1) Adolescent self-report limits scope of data that can be collected.  (2) 2 months reporting period may be too narrow, and the study may benefit from a longer period. |
| Depressed adolescents’ positive and negative use of social media [41]. | Teenagers or adolescents (11-18). | 23 subjects were interviewed. In 30-60 minutes, segments in semiformal fashion. | Demographic and social media use data taken to determine specific relationships between social media use in depressed youth. | Researchers set goals to determine how social media use affects depressive symptoms and behaviors among adolescents. Behaviors, such as stress posting and oversharing were directly linked to depressed symptoms. | (1) Some participants refused to offer supplemental information or were not able, which may lead to skewed or erroneous reporting  (2). Some participants may have been concerned with confidentiality  (3). Small sample size limits generalizability. |
| The impact and response to electronic bullying and traditional bullying among adolescents [43]. | Teenagers or adolescents (11-18 years). | Data is gathered from 97 students, and their responses are examined. | Social media presence and cyberbullying are correlated and investigated in the study. | The researchers look to explore the impacts and relevant responses to cyberbullying and contrast them with real-world bullying. Male instances of bullying were found to be more upsetting; however, higher levels of emotional distress were not clarified. Female and male forms of retaliation vary, with aggressive retaliation being common. | (1) The scenario used does not allow a clear distinction to whether all OA experiences are embarrassing.  (2) Some newer forms of communication, such as twitter, snapchat, and Instagram were not used.  (3) Sample size prevented correlations between victims’ gender and participant’s gender. |
| Applying artificial intelligence to explore sexual cyberbullying behavior [32]. | Young adults (19-25 years). | 374 young adults were sampled, classification trees were used as well as equation modeling AI^c^ to analyze data. | Young adults were sampled to discover specific behaviors linked to sexual cyberbullying. | Researchers wished to test the efficiency of cyberbullying preventing technologies in analyzing socially deviant behaviors linked to cyberbullying. The tool was successful in predicting levels of psychopathy and Machiavellianism. There was also a strong correlation between these features and sexual cyberbullying behaviors. | —^b^ |
| *Arguments online, but in school we always act normal*: the embeddedness of early adolescent negative peer interactions within the whole of their offline and web-based peer interactions [36] | Teenagers or adolescents (11-18 years). | 34 adolescents sampled through photoelicitation interviewing. | Students are interviewed to investigate the range of in-person and web-based negative peer interactions. | The goal of the study is to investigate how negative web-based and offline interactions are associated. It was found that participants generally only react in the same environment instead of changing environments. Those acting on the internet generally do not acknowledge events that have happened on the internet in person. | —^b^ |
| Sustainable cyberbullying detection with category-maximized relevance of harmful phrases and double-filtered automatic optimization [31]. | Teenagers or adolescents (11-18). | Researchers used phrase extraction, categorization, and relevance maximization to analyze relevant terms. Data was collected from school platforms. | Search technologies are used to filter text and identify cyberbullying terms and phrases. | The researchers looked to test the method currently being proposed and understand how the results produced differ from the baseline. | —^b^ |
| Online social support for young people: Does it recapitulate in person social support; can it help? [11] | Young adults (19-25). | 256 students were surveyed, and responses were analyzed | Both traditional and cyberbullying were investigated to determine effective methods. | Researchers looked to understand methods for circumventing the negative effects of cyberbullying and victimization. Social support was found to ameliorate the destructive effects of peer victimization. Web-based and in-person social support was shown to have advantages in different areas over one another | —^b^ |
| Classification and its consequences for online harassment: design insights from HeartMob [42] | Young adults (18+) | Interviews through the HeartMob system. Users were located via email list. | Subjects are studied for their perceptions of web-based social interactions in online communities and web-based harassment enabled by technological platforms. | Researchers wish to understand social misbehavior and ways to mitigate and moderate it. Normative and nonnormative behaviors are classified through previous theories and applied to the findings. It is discovered that labeling web-based experiences as harassment provides validation and allows bystanders to grasp the situation and act more accurately. | (1) Limitation of demographics (mostly white) because racism can be a significant factor.  (2). Lack of nonconforming and transgender participants. |
| The third person effect on Facebook: the significance of perceived proficiency [16] | Mixed | 503 participants are surveyed from a sample of Facebook users from Israel. | Data is analyzed from social networking platforms to contextualize cyberbullying. | Researchers attempt to understand the motivations and perceptions of bystanders in relation to witnessing cyberbullying. The study finds that most users have an inflated sense of control over their lives. | —^b^ |
| Cyberbullying impacts on victims’ satisfaction with information and communication technologies: the role of perceived cyberbullying severity [44] | Adults (25+) | 115 victims were sampled with a 12-month reporting timeframe. Subjects were interviewed about their experiences. | Study investigates users’ perceptions of social platforms after being exposed to cyber harassment through social platforms. | The study aims to discover the long-term effects of cyberbullying on perceptions of the current social networking technologies. It was found that these cyberbullying events negatively impact the victim’s satisfaction with the related ICT platform. | —^b^ |
